# Focusing super resolution on the cytoskeleton

**DOI:** 10.12688/f1000research.8233.1

**Published:** 2016-05-25

**Authors:** Eric A. Shelden, Zachary T. Colburn, Jonathan C.R. Jones

**Affiliations:** 1 School of Molecular Biosciences, Washington State University, Pullman, WA, USA

**Keywords:** microscopy, live cell imaging, cytoskeleton

## Abstract

Super resolution imaging is becoming an increasingly important tool in the arsenal of methods available to cell biologists. In recognition of its potential, the Nobel Prize for chemistry was awarded to three investigators involved in the development of super resolution imaging methods in 2014. The availability of commercial instruments for super resolution imaging has further spurred the development of new methods and reagents designed to take advantage of super resolution techniques. Super resolution offers the advantages traditionally associated with light microscopy, including the use of gentle fixation and specimen preparation methods, the ability to visualize multiple elements within a single specimen, and the potential to visualize dynamic changes in living specimens over time. However, imaging of living cells over time is difficult and super resolution imaging is computationally demanding. In this review, we discuss the advantages/disadvantages of different super resolution systems for imaging fixed live specimens, with particular regard to cytoskeleton structures.

## Introduction

Visualizing proteins indirectly in cells and tissues at the light microscopic level using antibodies conjugated to fluorochromes revolutionized the field of cell biology 40 years ago. In the late 1980s and early 1990s, the use of commercial, user-friendly, confocal microscopes in combination with digital image acquisition systems dramatically improved the image quality of fluorescently labeled specimens and made image capture easier by avoiding the vagaries of the dark room. The technical benefits of confocal over “conventional” microscopy include the removal of the out-of-focus glare that interferes with what the imager really wishes to observe and an increase in specimen contrast. More recently, the ability to follow tagged molecules in live cells using both conventional and confocal fluorescence microscopy and the development of molecular biosensors has allowed cell biologists to study the dynamics of individual proteins and protein complexes with high precision. Conventional techniques used for light microscopy can achieve resolutions of up to ~100 nm in the image plane, as first detailed by Abbe ^1^, and about twice this value along the focal axis ^2^. In practice, most authors consider the resolution of commercially available microscopes to be ~200 nm in the image plane and ~500 nm in the axial dimension. So called “super resolution” is achieved with techniques that allow resolution beyond the diffraction limit of conventional optics ^3^. The term super resolution as applied to microscopy has been in use since at least as early as the 1960s ^4^. Resonance energy transfer ^5^, near field scanning optical microscopy ^6^, dual objective (4Pi) microscopy ^7^, total internal reflection microscopy ^8^, single molecule fluorescence localization ^9^, expansion microscopy ^10^, and several other strategies represent successful efforts to obtain structural or positional information from biological specimens at resolutions better than those afforded by conventional microscopy. However, many of these approaches are technically demanding or present significant limits to the type of specimens that can be examined. The last decade has seen the development of methods that closely resemble more familiar far-field and laser scanning confocal microscopy but allow direct visualization of subdiffraction size structures in fixed and living specimens. Current super resolution techniques provide resolutions of less than 10 nm in the image plane ^11^ and ~20 nm in the z-axis. The improved resolution offered by these methods has produced breathtaking images of the nuclear pore complex ^12, 13^, the tubular walls of microtubules ^14^, and many other structures.

Although the potential and allure of super resolution methods are indisputable, they also present new challenges to image acquisition, storage, and interpretation. For example, changing the resolution of an image from 200 nanometers to 20 nanometers in the image (xy) plane, while maintaining a fixed field of view, results in a 100-fold increase in image size. Extending these calculations to the third dimension, multispectral imaging and time (for live cells) reveals the potential extent of demands that super resolution microscopy can place on specimens and fluorophores as well as imaging and data processing hardware. Moreover, although computer and imaging technologies have advanced to the point where accumulating these data is feasible, human involvement is currently still needed to identify regions of interest for examination and analysis. Each of the methods used to achieve super resolution imaging also offer unique technical strengths and weaknesses to cell biologists. We direct the reader to several excellent recent reviews that provide an overview of the capabilities, advantages, and disadvantages of a variety of super resolution procedures in tabular form ^15– 18^.

Despite the inherent challenges, the use of super resolution microscopy is beginning to make an impact on a wide variety of biological topics. Among the subjects most likely to benefit from the application of super resolution imaging is the study of the cytoskeleton and its associated structures. Unlike many other cellular components, cytoskeletal filaments form anastomosing networks of fibers smaller than the resolution of conventional imaging methods. Techniques allowing enhanced resolution imaging of cytoskeletal structures, especially in live cells, are already advancing our knowledge of their formation and function. For example, super resolution has been a boon to investigators studying cytoskeletal rearrangements in bacteria ^19, 20^ and yeast cells ^21^, which have generally been too small to approach with conventional diffraction-limited imaging methods. Below, we briefly discuss the major methods for achieving super resolution as well as their strengths and weaknesses with emphasis on recent work in which these methods have been applied to address biological questions involving the cytoskeleton.

## Localization microscopy

Stochastic optical reconstruction microscopy (STORM ^22^), photoactivated localization microscopy (PALM ^23^), fluorescence photoactivation localization microscopy (FPALM ^24^), and a growing number of related methods are techniques where fluorescent specimens are examined by activating a limited set of fluorophores at a time which must be separated by distances greater than the resolution limit of the microscope. A diffraction-limited image of the fluorophores is captured and the position and intensity of each fluorophore calculated at subdiffraction precision. Activated fluorophores are deactivated, and a new set of fluorophores is activated and imaged. After many images are collected, a completed super resolution image is calculated. The techniques differ in the type of fluorophore used. For example, PALM and FPALM are used to image expressed photoactivatable fluorescent proteins and fusion proteins ^23, 24^, while STORM is used to create images of fluorescent dyes and tags that can be switched between fluorescing and non-fluorescing states ^22^. In practice, these methods can produce the highest resolution of the available diffraction-unlimited techniques when applied to biological specimens, with two-dimensional resolutions of less than 20 nm frequently reported ^12, 25– 27^. However, overall image resolution and quality increase with the number of photons captured and fluorescent molecules examined. Therefore, these methods are limited by the time required to obtain a sufficient number of images – often tens of thousands – needed to create a final super resolution image. In addition, many conventional fluorophores and fluorescent proteins are not suitable for localization microscopy ^14, 28^, making some techniques such as multicolor staining methods challenging. Localization methods also require that fluorescent molecules or proteins within the specimen be detected individually, complicating the application of these techniques to densely labeled three-dimensional cytoskeletal arrays or arrays contained within thick samples exhibiting autofluorescence. To address this, some investigators have incorporated total internal reflection fluorescence illumination (TIRF), two photon illumination, or light sheet illumination to limit the volume of a specimen under inspection ^23, 29– 31^. However, these implementations increase the complexity of the instrumentation required. Despite these limitations, a large number of studies encountered in our review of current literature employ localization techniques. This may be because of both the superior resolution of the methods and the relatively simple hardware requirements which have allowed many investigators to build their own STORM or PALM/FPALM imaging systems. In addition, fluorophores, fluorescent proteins, buffering agents, and illumination strategies are being actively developed to extend localization to both multicolor labeling and other fluorescent imaging tasks (see 28, 32– 35 for representative reviews).

Among the most novel and dramatic discoveries made using localization methods is the periodic distribution of actin filaments and associated cytoskeletal proteins in axons first visualized using STORM imaging ^36^. The periodicity of this structural feature of neurons is below the resolution limit of conventional microscopy and was overlooked in thin section electron micrographs ^37, 38^. Other studies have further exploited single molecule/protein imaging to analyze the development and regulation of these arrays ^39, 40^. STORM and PALM methods are also being applied to the study of the structure of adhesion complexes such as hemidesmosomes ^41^, intercellular adherens junctions ^42, 43^, and the less well-defined adhesions formed by leucocytes ^44^, as well as actin cytoskeletal rearrangements that occur during endocytosis ^45^ and bacterial host cell invasion ^46^. In the case of adherens junctions, STORM analyses indicate that E-cadherin exists in clusters at sites of cell-cell interaction rather than as the “solid” belt typically observed by non-super resolution methods ^42, 43^. Similarly, dual-color PALM studies suggest that paxillin and vinculin form functionally distinct non-overlapping nanoaggregates in focal adhesions that are not detectable using conventional imaging methods ^47^.

Microtubules, 24 nm diameter tubes composed of protofilaments, are sparsely distributed at the edge of cultured cells and are often used as proof-of-concept targets by developers of super resolution imaging methods ^48– 51^. STORM and PALM imaging are beginning to provide new details regarding the function and organization of the microtubule cytoskeleton. For example, these techniques have been used to study the organization of centrosomal proteins in intact cells, the architecture of microtubules underlying the movement of organelles within living cells ^52^, and the interaction of kinesin motor proteins with microtubules in neuronal processes ^53^. A variant of PALM imaging has also been used to study the structure of EB1 at the distal tip of growing microtubules ^54^, and PALM has made possible the visualization of FtsZ, the bacterial homolog of eukaryotic tubulin, in distinct polymeric arrays in prokaryotes ^20, 55^.

Although relatively few studies have examined intermediate filament arrays using super resolution imaging, STORM has been used to investigate keratin, plectin, and integrins in hemidesmosomes formed by cultured keratinocytes ^41^. Additionally, desmin, a cytoskeletal protein mutated in clinically important cardiomyopathies, has been visualized using dual color PALM microscopy in cultured cardiomyocytes ^56^. In the latter study, the authors report a 10-fold increase in the resolution of desmin protein aggregates and filaments over non-super resolution light microscopic methods. More importantly, their super resolution images reveal that both mutant and wild-type desmin proteins are incorporated into the same filament, suggesting the possibility that changes in the mechanical properties of a “mixed” filament might be the cause of disease ^56^.

## Structured illumination microscopy

Structured illumination microscopy (SIM) improves the resolution of light microscopy by illuminating a specimen with a defined regular pattern of diffraction-limited light and dark bands which create Moiré patterns when combined with the structure of a specimen ^57^. An image of the resulting interference pattern is created and recorded. The illuminating pattern is then rotated and further images captured. Finally, an image with improved resolution is calculated from the combined rotation series. Because the illuminating pattern is generated using wide-field optics, the technique exposes a specimen to illumination levels that are comparable to other wide-field microscopy methods (although multiple images must be captured for each view of the specimen). While many implementations of localization microscopy use total internal reflection illumination and are therefore limited to observation of structures within ~100 nm of an optical surface, SIM can resolve structures many microns deep within a specimen. SIM can also be used with any fluorescent probe and, unlike localization methods or laser scanning methods such as stimulated emission depletion microscopy (STED), complete images of a specimen are obtained at speeds determined by camera sensitivities. SIM is therefore one of the least phototoxic and most rapid methods for obtaining enhanced resolution images and has been used extensively in studies of living cells. A three-dimensional version of structured illumination allows for improved resolution in the z direction and has been used to visualize cytoskeletal structures in three dimensions. Both of these applications are discussed below.

The resolution achieved with SIM is generally only twofold greater than that offered by conventional microscopy ^57^, although some implementations allow SIM to achieve lateral resolutions as high as 50 nm ^58, 59^. However, even a twofold increase in resolution, combined with the power of multispectral fluorescent labeling methods, has allowed investigators to observe previously unresolved features of a wide variety of cytoskeletal structures. For example, SIM has been used to characterize the distribution of microtubules and associated structures in a variety of specimens that conventional diffraction-limited imaging methods have been unable to resolve well, including the neuromuscular junction ^60^, platelets ^61^, centrosomes ^62, 63^, and protists ^64^. SIM has also facilitated studies investigating the structure of striated muscle ^65, 66^ as well as actin and myosin filament organization in non-muscle cells ^67– 70^. Intermediate filament architecture and associated junctions have also been examined using this method ^71, 72^. Interestingly, although the localization-based imaging methods described above achieve higher resolution than SIM, the pointillized appearance of images produced by localization microscopy can obscure fine structural detail that may be visible using SIM. For example, SIM has revealed that focal adhesions comprise linear subarrays ^73^, a feature not readily visible in images published by investigators using interferometric (i)PALM to analyze the axial distribution of focal adhesion components ^74– 76^.

Finally, because SIM can be applied to microscopy of any fluorescent probe, it can be readily used for multispectral studies using conventional fluorophores. SIM has been used to examine cytoskeletal structures in triple-labeling studies of adherens junctions ^77, 78^, neuronal spines ^79^, centrioles ^80^, kinetochores ^81^, and endosomal vesicles ^82^. For example, SIM analyses of VASP, zyxin, testin, and other proteins surprisingly indicate that these tension-regulating proteins are likely recruited to adhesion junctions independent of the core adhesion complex ^78^.

## Stimulated emission depletion microscopy

STED, reversible saturable optical fluorescence transitions microscopy (RESOLFT), and related techniques excite fluorophores in a diffraction-limited spot by a focused laser. Outlying fluorophores are converted to a non-fluorescent state by illumination with a second (depletion) laser in a manner that leaves a central, subdiffraction limited area of fluorophores unconverted ^83– 85^. The remaining still-fluorescent fluorophores are detected to create an image with resolutions far greater than those provided by conventional imaging methods. Some implementations of these techniques have yielded resolutions in biological specimens of <50 nm in the image plane and 150 nm in the axial dimension ^86^. Common implementations of STED are technically demanding and the depletion light energies are substantially greater than the illumination intensities required by other super resolution imaging methods. However, STED and similar systems closely resemble laser scanning confocal microscopes already employed by many investigators, and these methods can be applied to most commonly available fluorophores. Unlike localization methods and SIM, STED does not require calculations to generate enhanced resolution images, and image resolution can be readily varied by changing the raster scanning patterns used to visualize a specimen. Scanning rates achieved by STED are suitable for imaging of live specimens ^87, 88^, but the required depletion energies currently make extended live cell imaging a challenge. However, super resolution imaging can also be interchanged with conventional confocal scanning approaches simply by turning the depletion laser on or off.

**Figure 1.**
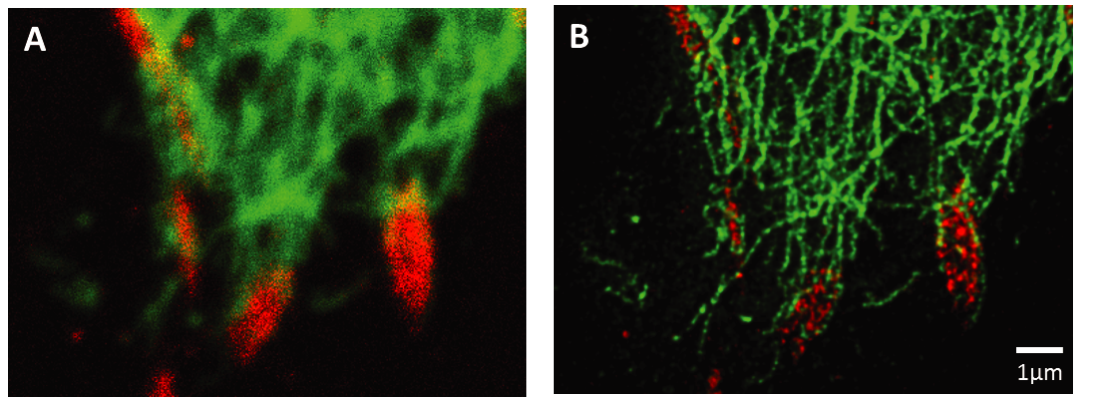
Association of paxillin (Alexafluor 555, red) and vimentin (Oregon green 488, green) at focal adhesions located at the leading edge of a migrating A549 lung cell. Conventional confocal imaging is shown in **A** while STED imaging of the same area is shown in **B**.

STED has been used to visualize the periodic ring structure of actin in neurons ^89^ and actin dynamics in dendritic spines of neurons in living brain tissue ^90^. Others have used STED to examine the actin-like MreB protein in bacteria ^91^ and the reorganization of actin filament arrays during activation of natural killer cells and T cells ^92, 93^. It has allowed the visualization of myosin mini-filament formation in mammalian non-muscle cells ^94^ and insight into the regulation of the actin cytoskeleton by intracellular signaling proteins ^95, 96^. Microtubules have been examined using STED in primary cilia ^97^, and the interphase microtubule array has been examined in muscle and non-muscle cells ^98, 99^. Finally, several groups have utilized STED microscopy to visualize elements of the intermediate filament network, including vimentin ^100, 101^, keratin ^102^, and nuclear lamins ^103^. In our laboratory, we have used STED to assay the relative localization of vimentin and focal adhesions in cultured epithelial cells. Whether vimentin interacts with focal adhesions has been controversial for years ^104, 105^. However, STED not only provides images with more detail than can be obtained using conventional confocal microscopy but also reveals a distinct pattern of organization of paxillin within a focal adhesion ( Figure 1). Moreover, in the STED image, vimentin filaments clearly wrap around each focal adhesion, an interaction that is not apparent in the confocal image.

## Live cells

The ability to study proteins in cells in the living state is one of the most significant advantages of light microscopy over other methods of analysis. However, as has been noted by many others (see 106– 108 for representative reviews), obtaining images of living cells that are doing something other than dying on the microscope is difficult and involves balancing the competing requirement of image quality and cell health. In addition, the study of cytoskeletal dynamics often requires rapid image acquisition, increasing the light exposure of live cells over time. Achieving resolutions greater than the diffraction limit of conventional microscopy ultimately requires capturing larger numbers of photons from smaller areas of live cells than is necessary for conventional resolution images, an effort that can clearly compromise the integrity of cells under examination ^108^. Fluorescently labeled cytoskeletal filaments are also notoriously photolabile ^109^. To date, many investigators of the cytoskeleton in live cells have combined super resolution microscopy of fixed cells with wide-field or confocal imaging of live cells ^39, 44, 45, 52, 53, 64, 68, 69, 77, 79, 98, 110– 113^ or have obtained single images of live cells using super resolution methods ^56, 89^. Although the latter avoid potential artifacts induced by fixation and immunostaining, this approach does not address dynamic changes in cell architecture.

The majority of publications in our survey that examined dynamic changes in cytoskeletal architecture employed SIM. Observations of the cytoskeleton in live cells have been made over time scales ranging from seconds to tens of minutes using this approach. The twofold resolution enhancement offered by SIM has allowed investigators to examine in previously impossible detail the growth of microtubules in living plant cells ^114^, actin retrograde flow in cultured insect cells ^115^, and the reorganization of cytoskeletal arrays in dividing yeast and bacterial cells ^21, 91^. Two-color applications of SIM in living cells have been used to demonstrate heterotypic assembly of myosin II isoforms ^67^, the spatial relationship between myosin IIA and alpha actinin ^116^, the clustering of receptors in adherens junctions ^117^, and that vimentin intermediate filaments move bi-directionally along microtubules ^118^.

Although capturing clear images of closely apposed structures using localization methods requires accumulation and processing of thousands of images over periods of many seconds to minutes, localization methods have also been applied to the study of rapidly changing cytoskeletal structures in live cells by sacrificing some image clarity to improve temporal resolution. For example, PALM has been used to quantify the addition and loss of individual paxillin proteins at focal adhesion sites with a spatial resolution of 60 nm and temporal resolution of 25 seconds ^119^. Proof-of-principle studies have also demonstrated that PALM can be used to visualize dynamic changes in fluorescent actin filaments over brief intervals ^120^ and the movement of fluorescently labeled endosomes along microtubules in living axons ^121^. In addition, PALM imaging platforms have been exploited by several groups that have followed changes in localization of single fluorescent cytoskeletal proteins over time, either alone or in combination with PALM, STORM, or STED techniques ^122– 125^. This appears to be a powerful, multimodal approach that can place the movement of individual proteins in the context of cytoskeletal architecture.

STED employs depletion lasers at energies that are largely incompatible with extended viewing of live cells, and, not surprisingly, we encountered few publications where this method has been used to image cytoskeletal arrays *in vivo*. Nonetheless, STED has great potential for imaging structures within complex tissues and has been used in a seminal study of actin dynamics in living neurons within brain tissue ^90^. Single images of microtubules within living cells have also been obtained at 60 nm resolution using STED ^86^. STED has also been applied to imaging using multiphoton excitation ^126– 128^ and total internal reflection microscopy ^129^. These methods limit light exposure to a sample and offer further potential for the application of STED illumination to live cells. We anticipate that as these complex instruments become more widely available, we will see an increase in the number of investigators employing them for studies of living cells.

## The third dimension

Many initial implementations of super resolution methods did not provide an increase in resolution in the third, or axial, dimension of the microscope (see 18 for review). However, recent modifications to these methods have achieved impressive resolution enhancements in the third dimension. Three-dimensional SIM doubles the resolving ability of the light microscope in all dimensions while retaining its ability to obtain images rapidly with low light exposures ^130^. Although many studies have captured Z-stacks of images using super resolution methods and generated extended focus images from them, relatively few studies have analyzed the cytoskeleton in three dimensions using super resolution microscopy. However, three-dimensional SIM has been used to resolve actin filament arrays and microtubules in three dimensions using both fixed and live cultured cells ^113, 116, 130, 131^ as well as to study the structure of centrosomes ^63, 80^ and kinetochores ^81^. Additionally, this method has also been used to visualize the three-dimensional organization of FtsZ in dividing bacteria ^132^. Modification of STORM and PALM imaging platforms can achieve axial resolution of up to 20 nm by introducing axial astigmatism into the optical path ^133^ and by generating interference patterns from images obtained with paired, opposed objective lenses ^134^. Three-dimensional STORM and PALM have been used to study the movement of organelles along microtubules in live cells and the formation of FtsZ ring structures in live dividing bacteria ^20, 52^. A STORM imaging method employing axial astigmatism and dual objectives has been used to show that sheet-like cellular extensions in cultured cells contain two distinctly separate actin filament arrays each with unique patterns of actin filament organization ^134^. The development and regulation of these previously undetected cytoskeletal arrays have been further analyzed using similar approaches in normal cell movement ^112, 116^. iPALM can resolve structures less than 20 nm in diameter in three dimensions ^27^. The technique has recently been used to dissect the organization of cytoskeletal proteins associated with matrix adhesion devices termed focal adhesions at an unprecedented level of detail ^74– 76^. Impressively, the technique was able to resolve not only vertical stratification enriched for specific cytoskeletal components but also the polarized orientation of the N- and C-terminal ends of talin within focal adhesions of intact cells. Finally, STED microscopy has also been modified by the addition of dual depletion patterns, one oriented in the image plane and the other in the axial dimension, allowing for increases in resolution in the third dimension of up to 125 nm ^135^. STED and RESOLFT have also been extended to the third dimension using a dual objective imaging strategy ^136^.

Presently, most work using super resolution imaging has been conducted using single cells cultured on optical surfaces. However, previous work has shown that cell morphology and behavior can be dramatically altered in three-dimensional environments ^137, 138^. To date, the literature examining the cytoskeleton in cells *in situ* is largely limited to proof-of-principle studies. However, these efforts demonstrate the growing potential of super resolution methods. For example, STED has been used to view the dynamics of actin filament arrays within live neurons in 350 μM thick brain slices ^90^, and three-dimensional SIM has been used to visualize actin arrays in developing Drosophila ^115^. In other studies, labeled nuclear histones have been visualized in cells within 150 μM cell spheroids by combining single molecule localization methods with planar illumination ^139^. Both planar and multi-photon illumination methods have been coupled with structured illumination to view green fluorescent protein (GFP) expressed in living nematodes ^140, 141^. These methods improve visualization by restricting effective illumination to a single plane of interest within thick specimens. While these latter studies examined structures other than the cytoskeleton, these methods show promise for the analysis of the cytoskeleton at subdiffraction resolutions *in situ*.

## Summary

Many of us have been fortunate to work as cell biologists during two major revolutions in imaging technology: the development of fluorescent proteins as tools for biologists and the development of confocal microscopy, which extracts clear, in-focus images in which the contaminating blur of out-of-focus structures has been removed. The astonishment and wonder with which we now view images created by super resolution microscopy suggest that we are experiencing yet a third revolution in the technology available to cell biologists. It is likely that for dual and triple labeling studies of cultured cells in two and three dimensions, as well as studies of bright, relatively slow-moving structures in live cells, super resolution imaging will soon replace confocal microscopy as the state of the art in much the same way that confocal microscopy replaced conventional wide-field imaging in the 1980s and 1990s. However, each of the approaches used to achieve images at better than diffraction-limited resolution have strengths and weaknesses. Studies of rapid cytoskeletal dynamics in live cells and three-dimensional studies are likely to present technical and biological challenges to practitioners of super resolution microscopy for some time. In addition, while the best super resolution light microscopic methods achieve resolutions of <10 nm, this is still 50–100-fold greater than the resolution afforded by electron microscopy ^142, 143^. Conventional fluorescence microscopes can also take advantage of a myriad of probes for physiological conditions and molecular interactions that have yet to be adapted to super resolution imaging methods. For the time being, no method addresses all possible experimental needs, and investigators will likely have to address the limitations of their super resolution instruments with complementary approaches involving conventional methods. Moreover, the dream of seeing individual protein complexes and their partners functioning in live cells within a complex three-dimensional organism remains unrealized. Nonetheless, we are seeing the development of instrumentation, computational methods, fluorescent probes, and novel methods at an amazing pace. We can only imagine what the next advances will be.
